# Introduction of amino groups into lignin and lignin model compounds: recent advances

**DOI:** 10.1039/d5ra05735c

**Published:** 2025-11-25

**Authors:** Veronika D. Badazhkova, Risto Savela, Reko Leino

**Affiliations:** a Johan Gadolin Process Chemistry Centre, Laboratory of Molecular Science and Engineering, Organic Chemistry Research Group, Åbo Akademi University FI-20500 Turku Finland reko.leino@abo.fi; b Turku PET Centre, University of Turku and Turku University Hospital FI-20520 Turku Finland; c Department of Chemistry, University of Turku 20500 Turku Finland

## Abstract

Research on lignin valorization has attracted considerable attention in recent decades. Lignin, a natural aromatic polymer, is a renewable and readily available source for producing new biobased chemicals and materials. For successful lignin valorization towards value-added products, development of efficient procedures allowing the introduction of new functionalities to its structure becomes essential. One of the approaches for chemical modification of lignin involves the introduction of amino groups. Lignin amination methodologies potentially provide aromatic amines that could be used as building blocks for the synthesis of different compounds, including pharmaceuticals and precursors for bio-based polymeric materials. Due to the complex structure of lignin, however, model compounds are commonly used for studying the reactivity and for developing lignin modification procedures. In several studies, it has been demonstrated that lignin model compounds imitating the most abundant lignin β-O-4 linkage can be converted to nitrogen containing aromatic compounds with various potential applications. However, it has also been observed in several cases that the developed amination procedures only have functioned on the model compounds but not on extracted lignin polymer. Overall, in recent years, several procedures have been developed for amination of lignin model compounds and lignin, allowing materials containing primary, secondary and tertiary amines to be obtained, for example the Mannich reaction, silylation and epoxy functionalization. The chemical modification and analysis of lignin are nevertheless hampered by its complex and inconsistent structure, varying significantly depending on the source and method of isolation. While the bulk of currently available lignin is present mainly in the form of Kraft lignin with condensed structure and low solubility, new efficient methods could facilitate switching to lignin-first approaches, preserving its original structure and improving the processing towards value-added products.

## Abstract 2

Pathways to introduce amino groups into lignin-derived monomers and technical lignin are reviewed.

## Graphical abstract

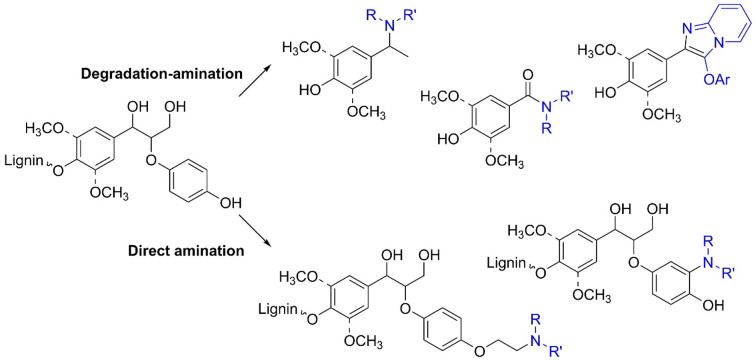

## Introduction

Lignin, the second most abundant natural polymer in the world, is a highly promising renewable feedstock for producing new chemicals and materials.^1^ The industrial production of lignin reaches up to 100 million tons per year. However, a major part of the lignin produced by the paper industry is utilized in combustion for energy production, and only 1–2% of the isolated lignin is used for other applications as value-added products.^2^

**Fig. 1:**
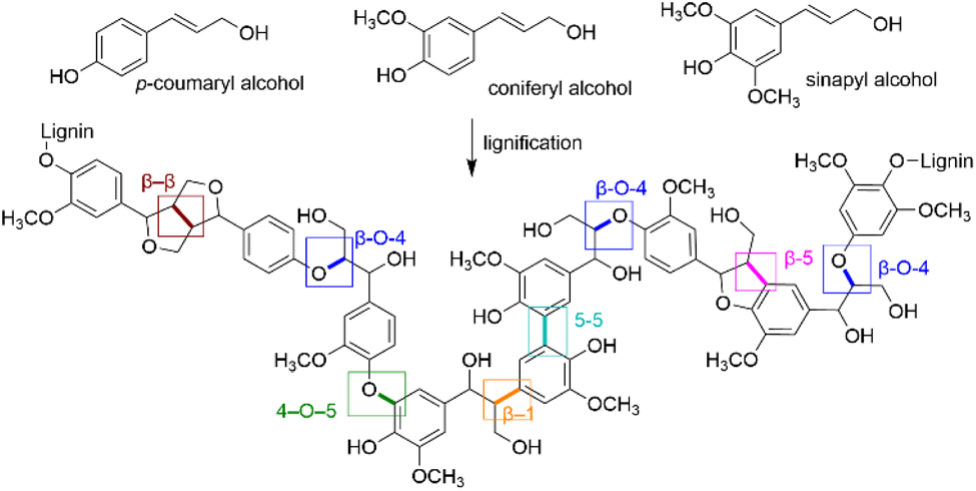
Illustration of a representative lignin structure containing the most common linkages formed between the monomeric units.^4^

The chemical structure of lignin is often presented as a crosslinked network of primarily three phenylpropane units, *p*-coumaryl, coniferyl, and sinapyl alcohols, also known as monolignols. During lignification – lignin polymerization processes in a plant cell – various inter-unit linkages are formed within the polymeric structure. The most common linkages in lignin structures are β-O-4, β-5, 5-5, 4-O-5, β-β and β-1 linkages (Fig. 1). In native lignin, β-O-4 type is the most abundant linkage.^3^ The high carbon content and the aromatic structure of lignin then render it as a renewable precursor for producing aromatic compounds.

Due to its thermal degradation and mechanical properties, the direct application of lignin is limited, yet possible, for example, as antioxidant, UV stabilizer or additive for plastics.^5^ The main challenge in lignin valorization is its structural complexity and heterogenity.^6^ The structure, molecular weight and physical properties of lignin may vary considerably, depending on both the source and the isolation process.^7^

In recent decades, the research on chemical modification of lignin has rapidly developed, exploring the possibilities of lignin valorization with preservation of its macromolecular nature, as well as the degradation of lignin into smaller molecular components to obtain value-added products.^4,8–10^

The surface of extracted lignin is rich with phenolic (1.2–2.0 mmol g^−1^) and aliphatic (4.8–5.9 mmol g^−1^) hydroxyl groups that can be used for introducing new functionalities to its structure.^5,11^ In several studies on chemical modification of lignin, it has been shown that various oxidation, degradation, alkylation and amination procedures can be applied to both lignin model compounds and extracted lignin.^5^

In industry, amines are important building blocks for the synthesis of a wide range of products, including polymers, agrochemicals and pharmaceuticals.^12,13^ Consequently, development of amination procedures is a key to expand also the product pool of lignin-based chemicals.^14^ This short review summarizes the recent research on amination of lignin model compounds and extracted lignin by utilizing hydroxyl groups in the lignin structure, with β-O-4 motif bearing aliphatic hydroxyl groups as the major target.

## Amination of lignin model compounds

Due to the structural complexity and heterogeneity of lignin, model compounds representing different lignin functionalities are commonly used for studying the reactivity and potential valorization approaches. The formation of low molecular weight compounds is often observed in lignin degradation studies. Thus, the use of model compounds allows to simplify the analytical and procedure development processes, while at the same time potentially providing methods for valorization of lignin degradation products.^15^

### Amination of lignin-derived monomers

**Scheme 1:**
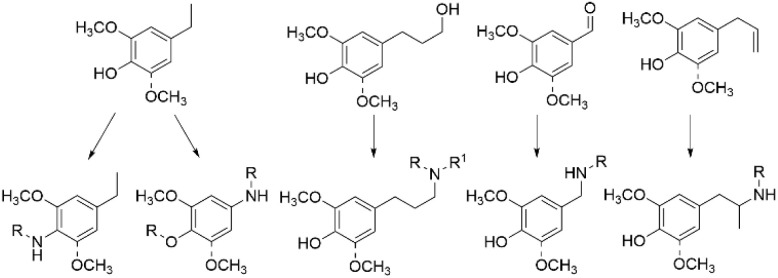
Amination of lignin-derived monomers obtained *via* RCF of lignin.

Amination of monomeric and dimeric compounds obtained by different lignin depolymerization strategies can be considered as valuable strategy for lignin valorization. Depending on the depolymerization approach, various phenolic products can be obtained from lignin and utilized for the synthesis of fine chemicals.^15,16^ For the transformations of lignin-derived monomers into animated products, various heterogeneous and homogeneous approaches can be used promoting the sustainable conversion and production of nitrogen-containing fine chemicals, including alkyl and aryl amines (Scheme 1).^17–21^ The possibilities of valorization of lignin-derived monomers towards aminated products have been frequently studied and this topic is sufficiently covered in literature elsewhere.^17–24^

### Catalytic amination of β-O-4 model compounds

**Fig. 2:**
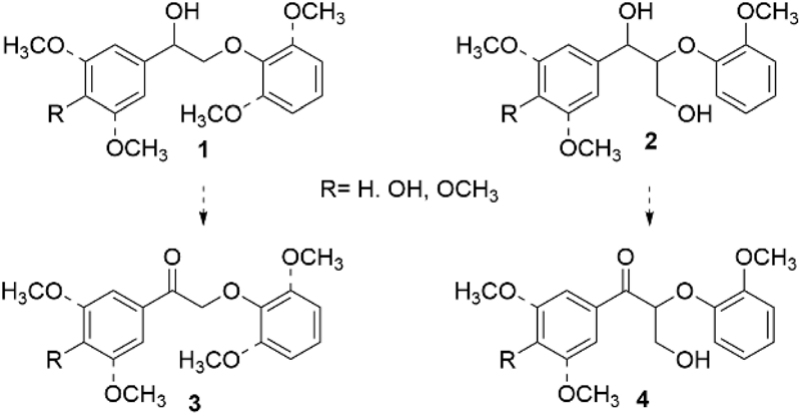
Representative structures of common β-O-4 model compounds.

β-O-4 model compounds, representing the most abundant lignin linkage, are frequently used in lignin valorization research.^25^ Specifically, 2-phenoxy-1-phenylethanols (1) and 2-phenoxy-1-phenyl-1,3-propanediols (2), bearing methoxy substituents in the aromatic rings to imitate original lignin structure, have been used as model compounds for development of various modification procedures, including amination. Depending on the catalytic system selected, for some reactions, 2-phenoxy-1-phenylethanones (3) and 3-hydroxy-2-phenoxy-1-phenyl-1-propanones (4) obtained from the corresponding alcohols are utilized as β-O-4 model compounds (Fig. 2).

Mechanistically, the amination processes generally consist of several subsequent steps. When β-O-4 alcohols are used as the model compounds, oxidation of the benzylic hydroxyl group to ketone is commonly the initial step of the subsequent multistep reaction. The ketone, either formed in the reaction mixture or used as starting material, then undergoes C–O and/or C–C bond cleavage followed by C–N bond formation *via* different mechanisms, depending on the reaction conditions.

**Scheme 2:**
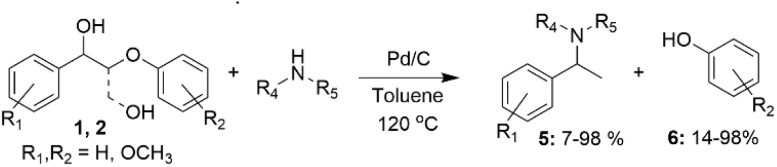
Conversion of β-O-4 lignin model compounds to benzylamines and guaiacol, catalyzed by Pd/C.

It has also been demonstrated that several lignin β-O-4 model alcohols 1 and 2 containing benzylic hydroxyl groups can be converted to benzylamines and phenols in the presence of Pd catalysts. Such transformations typically involve dehydrogenation of the benzylic hydroxyl group and hydrogenolysis of the β-O-4 bond in the lignin model alcohol, followed by reductive amination. Such reactions have been applied to several β-O-4 model alcohols with generic structures 1 and 2, using different amines and resulting in the formation of the corresponding benzylamines (5) and phenols (6) (Scheme 2).^26,27^ Presumably, compounds 1 and 2 undergo dehydrogenations of the benzylic hydroxyl groups, followed by C_β_–O_β_ bond cleavage for compound 1 and also C_β_–C_γ_ for compound 2, with subsequent imine formation and then its reduction to benzylamine. The yields of benzylamines obtained vary significantly, depending on the position of the methoxy substituents in the β-O-4 model alcohols and amines.

**Scheme 3:**
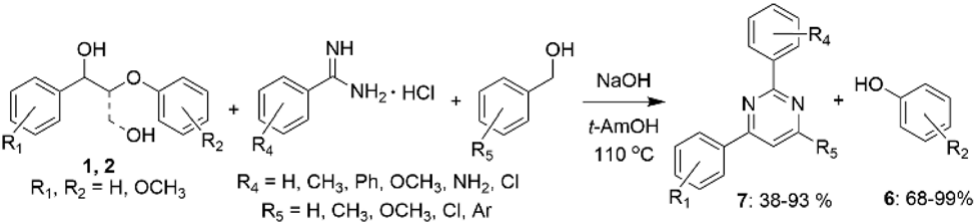
Conversion of β-O-4 lignin model alcohols to pyrimidines and phenols.

Li *et al.* have reported a one-pot multi-component cascade reaction for the synthesis of pyrimidines (7) from lignin β-O-4 model alcohols 1 and 2 (Scheme 3).^28^ According to the proposed mechanism, compound 1 undergoes conversion into acetophenone *via* dehydrogenation of benzylic hydroxyl group and C_β_–O_β_ bond cleavage. Acetophenone then undergoes cross-aldol condensation with arylaldehyde formed *via* dehydrogenation of the added benzyl alcohol, resulting in formation of chalcone that further reacts with benzamidine hydrochloride to form the final product 7. The reaction does not require the presence of transition metal and can be used for the synthesis of meridianin derivatives, potentially enabling the utilization of biomass for the synthesis of pharmaceutical precursors.

**Scheme 4:**
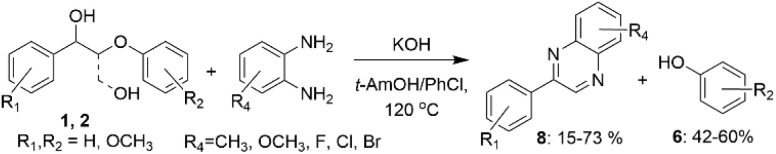
Conversion of β-O-4 lignin model alcohols to quinoxalines.

In other work, Li *et al.* described a one-pot transition-metal-free procedure allowing to obtain heterocyclic compounds from lignin model alcohols 1 and 2 and 1,2-diaminobenzenes (Scheme 3).^29^ The reaction consists of multiple cascade steps, including dehydrogenation-cleavage towards acetophenone intermediate that reacts with 1,2-diaminobenzene resulting in the formation of quinoxaline derivatives (8), including important biologically active compounds (Scheme 4). When β-O-4 lignin model alcohols with the general structure 1 were used, the yields of quinoxalines ranged from 48 to 73%. However, for the β-O-4 diols 2, the yields of the corresponding quinoxalines reached only 15%, explained by steric hindrance.

**Scheme 5:**
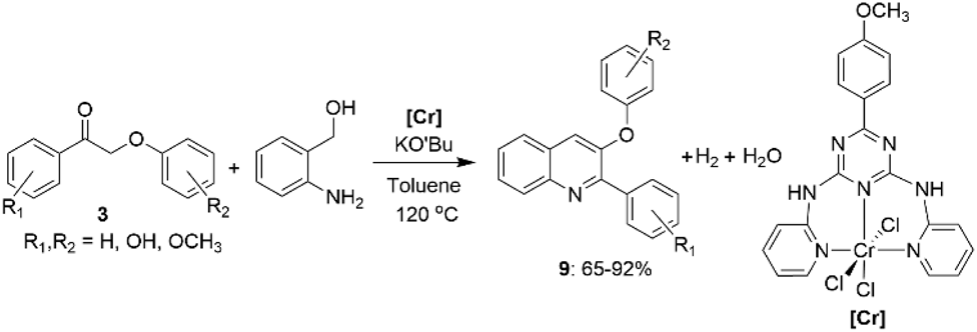
.Conversion of β-O-4 lignin model ketones to 2-phenyl-3-aryloxy quinolines.

Recently, it has been demonstrated that biologically significant nitrogen-containing heterocyclic compounds can be obtained from the lignin β-O-4 model ketones 3 using a chromium-based catalyst. Das *et al.* synthesized triazine-based Cr-NNN-pincer complexes to catalyze acceptorless dehydrogenative coupling reactions between 2-aminobenzyl alcohol and the lignin β-O-4 model ketones 3 (Scheme 5).^30^

**Scheme 6:**
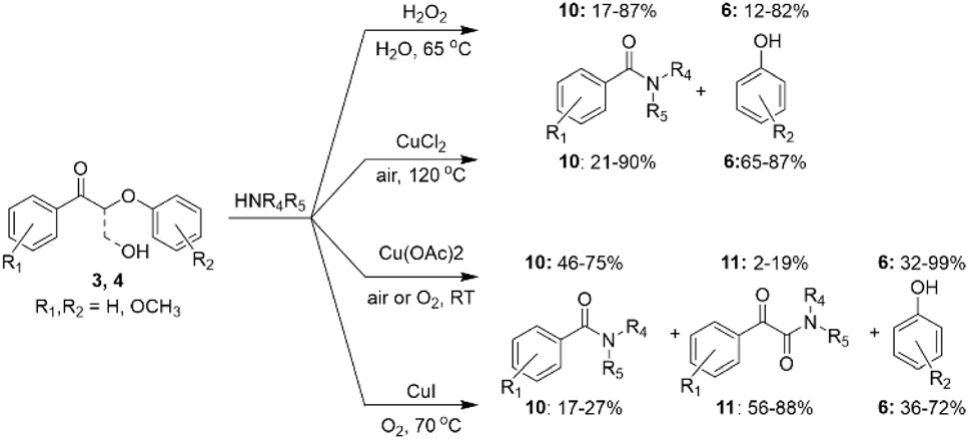
Cu-catalyzed amination of lignin β-O-4 model ketones.

The reaction subsequently undergoes dehydrogenation of 2-aminoaryl alcohol into corresponding aldehyde followed by aldol condensation between formed aldehyde and compound 3, followed by intramolecular condensation, C–N bond formation and nitrogen-heterocyclic ring construction steps, resulting in the formation of 3-phenoxy-2-phenylquinolines (9). Also, Cu-catalysts have been used in several studies for oxidative amination of lignin β-O-4 model ketones.^31–33^ Depending on the catalytic system and reaction conditions used, the amination resulted in the formation of different products (Scheme 6). Under air conditions and in the presence of CuCl_2_, the amination of β-O-4 model ketones 3 with aniline resulted in up to 90% yields of *N*-phenylbenzamides (10).^31^ Under oxygen atmosphere, CuI-catalyzed reaction of 2-phenoxyacetophenones (3) with piperidine led to the formation of α-keto amides in up to 88% yields, with no yield of amide reported. However, in the reactions of methoxy-substituted 2-phenoxyacetophenones (3) with piperidine, formation of both α-keto amides (11) as the major product and amides (10) as the minor products were reported.^32^ In Cu(OAc)_2_ catalyzed reactions, the amides (10) were found to be the major, while the α-keto amides (11) were minor products. However, the product ratio significantly depended not only on the rection atmosphere, but also on positions of the substituents in the substrate structure and the type of amine used for amination.^33^ Alternatively, it was shown that amides can be produced from β-O-4 model ketones under mild aqueous conditions using H_2_O_2_ instead of Cu-based catalyst in similar yields.^34^

**Scheme 7:**
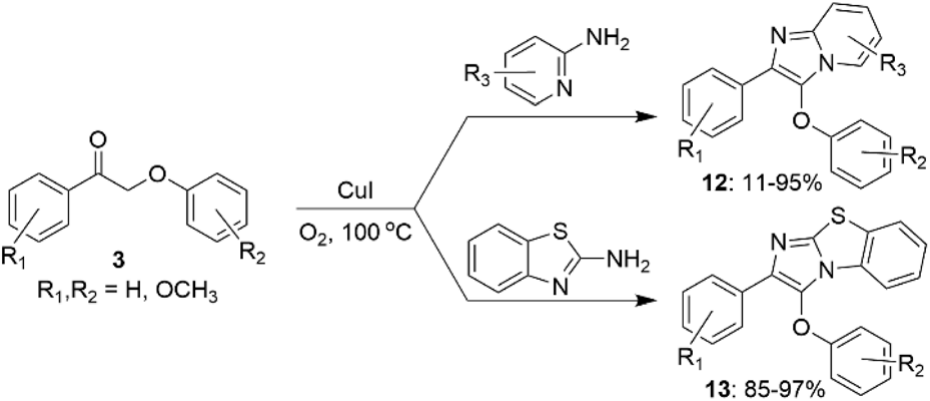
Conversion of β-O-4 lignin model compounds to imidazo heterocycles.

In other studies, CuI catalyst has been used for the synthesis of substituted imidazo heterocycles (12 and 13) from β-O-4 model ketones 3 (Scheme 7).

Compared to other Cu-catalyzed processes discussed above, C–O bond cleavage does not occur under the applied reaction conditions. This approach allows to obtain heterocyclic products (12, 13) with structures similar to some compounds used in drug design.^35^

**Scheme 8:**
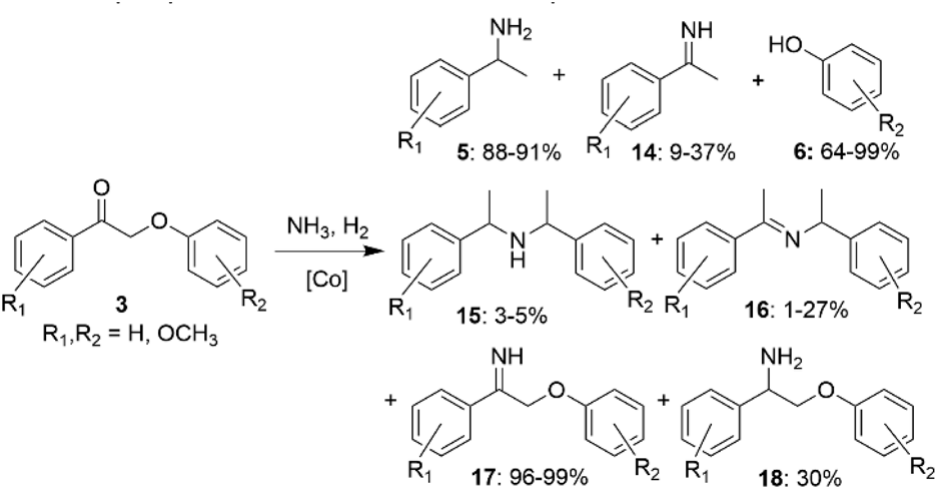
Co-catalyzed amination of β-O-4 lignin model compounds.

It has also been shown that heterogeneous Co-based catalysts can be used for amination of lignin β-O-4 model ketones.^36^ In a series of experiments using different Co-based catalysts, the predominant products in the reactions of lignin model ketones 3 with ammonia were benzylamines (5). For one of the catalysts, the reaction was highly selective towards imine aryl ether (17). In some cases, the formation of other imines (15, 16) and amines (14, 18) was also observed (Scheme 8). Selected Co-based catalysts allowed to reach high selectivities towards primary benzylamines that could be used as building blocks for further preparation of value-added products.

**Scheme 9:**
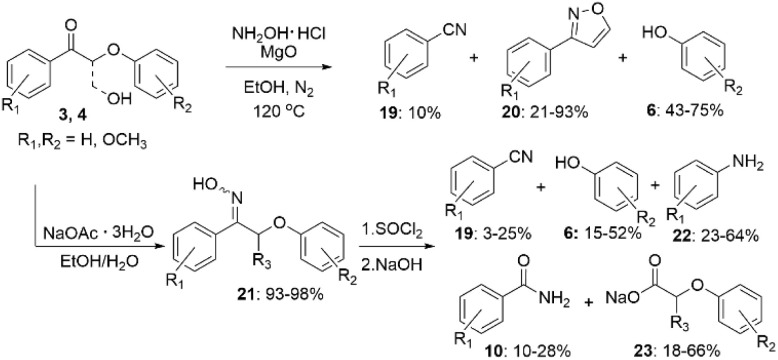
Lignin β-O-4 model compounds transformation *via* oxime formation.

New nitrogen-containing products can also be obtained by converting lignin β-O-4 model ketones to oximes for further transformations (Scheme 9). In the presence of MgO, the oxime formed as an intermediate in the reaction mixture, and could be further converted to aromatic nitriles (19) and isoxazoles (20).^37^ The MgO-catalyzed procedure was applied to dioxasolv birch lignin, resulting in bond cleavage and formation of isoxazole monomers.

Alternatively, the oximes (21) can be preliminary synthesized from the corresponding ketones (3, 4) and then used for subsequent degradation-amination. The reaction of β-O-4 oximes (21) with SOCl_2_ followed by NaOH addition resulted in a mixture of different products (10, 19, 22, 23) with N-containing functionalities.^38^

**Scheme 10:**
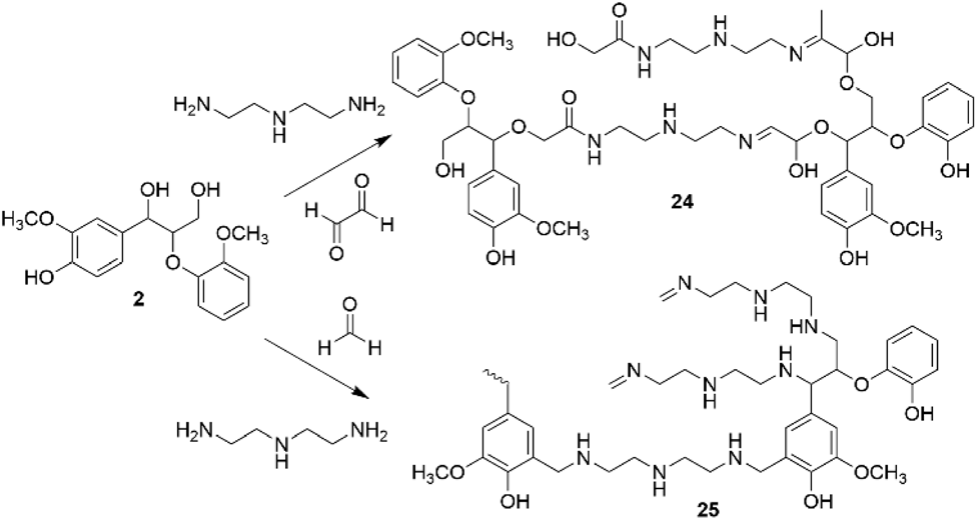
Cross-coupling amination reactions of lignin model compounds.

In recent work, Park *et al.* developed a one-step amination procedure of lignin model diol (2) with diethylenetriamine (Scheme 10).^39^

The reaction takes place *via* formation of methylene bridges and allows to convert lignin β-O-4 model compounds to crosslinked polyamines (24, 25). The reaction provides better understanding of crosslinking reactions by one-step amination which can be used for preparation of lignin-based materials.

**Scheme 11:**
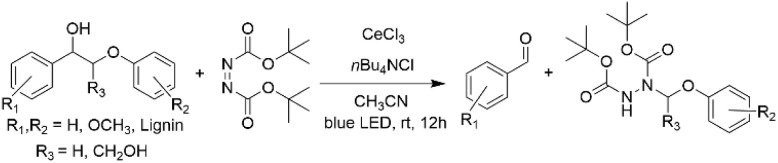
CeCl_3_-catalyzed photocatalytic amination of lignin β-O-4 model compounds and lignin.

Zhang *et al.* have demonstrated that lignin model compounds can be subsequently cleaved and aminated by tetrabutylammonium chloride using CeCl_3_-promoted photocatalysis.^40^ The benefits of this approach include the commercial availability of CeCl_3_, the possibility of re-use of the catalytic system over multiple reaction cycles and good reaction control by switching the external light on and off. The reaction was applied to several β-O-4 model alcohols (2, 3) providing aminated products (27) that can be further used for the synthesis of heterocyclic compounds (Scheme 11). The procedure was applied to extracted dioxane pine lignin primary consisting of β-O-4 and β-5 units, resulting in lignin depolymerization towards degradation products, including vanillin and hydrazine products.

## Lignin amination

For amination of lignin, various strategies utilizing different lignin functionalities have been used.

Due to the structural complexity of lignin and the typical range of functionalities, it is difficult to assess how the modifications take place mechanistically. Application of amination procedures first to lignin model compounds and analyzing the products formed can provide insights on the possible reaction pathways and new structures formed also in extracted lignin after modification. In addition, the data obtained from the analysis and characterization of the model compounds can often be used as reference for lignin characterization.

Different analytical methods and approaches can be used to evaluate the results after lignin amination.^41^ The combination of NMR spectroscopic techniques can be used for identification of different linkages in the lignin structure and reveal structural changes taking place in the reaction.^42^ FT-IR spectroscopy allows to determine the functional groups and their transformations.^43^ Elemental analysis provides information about the total nitrogen content in aminated lignin to reveal the extent of amination.^41^ X-ray photoelectron spectrometric (XPS) analysis can be performed to analyze the lignin surface and assess the chemical compositions of amino groups introduced.

### Amination by Mannich reaction utilizing the phenolic units

One of the most common approaches for introducing amino groups to lignin structures is the Mannich reaction, typically performed using formaldehyde and various amines, including dimethylamine, ethane diamine, diethylenetriamine, ethylenediamine, γ-polyglutamic acid, and other amines.^44,45^ Mannich reaction, in general, is the simplest and one of the most important fundamental amination reactions in organic synthesis.^46^ Already in 1956, Mikawa *et al.* studied the mechanism of Mannich reaction on lignin model compounds.^47^ In 1988 Brezny *et al.* reported the application of Mannich reaction to lignin for preparation of ion-exchanging lignin derivatives.^48^ Over the recent years, lignin pretreated with Mannich reaction has been used for formation of nanoparticles,^49^ preparation of bio-based fertilizers,^50,51^ azo-dyes,^52^ adhesives,^53^ and composites,^54^ formation of hydrogels,^55^ synthesis of polyurethanes,^56^ and for curing of epoxy resins.^57^

**Scheme 12:**
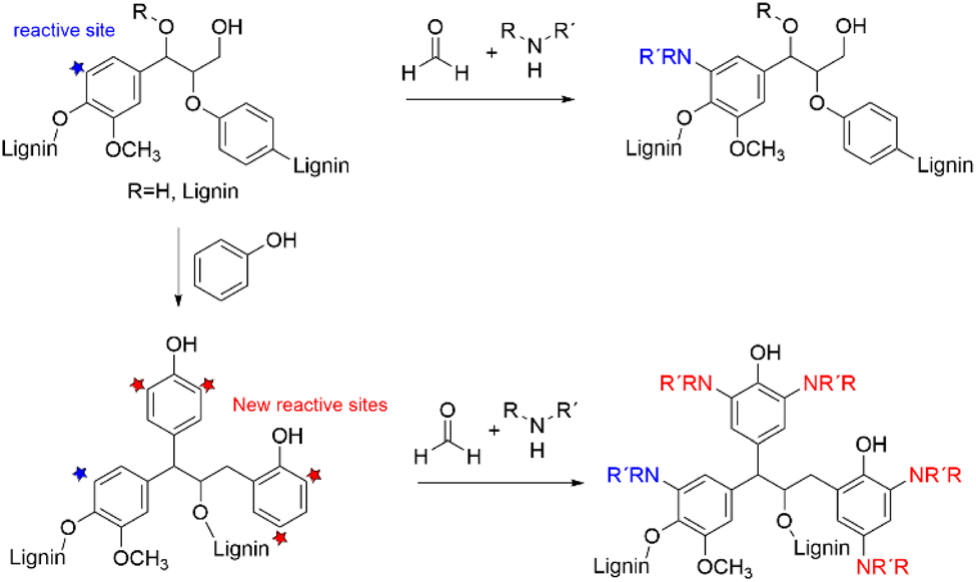
Amination of lignin and phenolated lignin by Mannich reaction.

After application of the Mannich reaction to alkali lignin with ethylenediamine, nitrogen contents reaching up to 5.81 wt% and with hexane-diamine up to 8.87 wt% have been obtained.^58^ To increase the nitrogen content, preliminary phenolation of lignin can be performed to introduce new phenolic units to the lignin structure to be aminated (Scheme 12).^59^ It has been shown that after the Mannich reaction the nitrogen content in phenolated lignin can reach 4.8 wt% compared to 2.5% in non-phenolated lignin.^46^ In other work, it was reported that the amount of active sites in phenolated lignin increased up to 8.26 mmol g^−1^ compared to 2.91 mmol g^−1^ in non-phenolated alkali lignin. The nitrogen content in phenolated alkali lignin has been reported as 10.13%.^50^

Despite that Mannich reaction is commonly used for lignin amination, it has several disadvantages. The Mannich reaction predominantly leads to the formation of tertiary amino groups over the primary and secondary ones, and it is difficult to control the actual reaction process. The reaction results in the formation of hindered polyamines with crosslinked structure and low solubility. Nitrogen content in the aminated lignin is typically relatively low, due to the low reactivity of extracted lignin and the limited amount of reactive sites in the phenolic groups that can be functionalized. All these factors restrict the potential application of aminated lignin.^41,60^ In addition, the reaction is commonly performed with the toxic and carcinogenic formaldehyde.^61^

### Amination by epoxy functionalization utilizing the phenolic hydroxyl groups

**Scheme 13:**
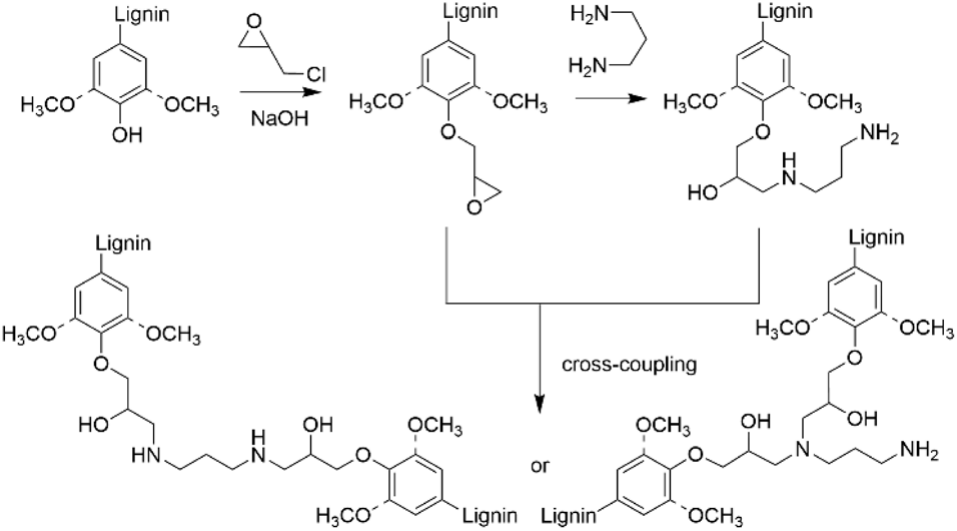
Lignin amination *via* epoxy functionalization.

To introduce amino groups to lignin structure, the phenolic hydroxyl groups in lignin can also be epoxy functionalized prior to the amination reaction (Scheme 13).^41^ In different studies it was demonstrated that Kraft lignin, having higher content of phenolic hydroxyl groups than native lignin, can be successfully epoxidized and cured using different amines, allowing to obtain lignin-based epoxy resins with different properties.^62–67^ After epoxy functionalization followed by amination, nitrogen content in aminated lignin reaching 6.95 wt% has been reported.^41^ Compared to the Mannich method, this approach allows to obtain lignin containing primary and secondary amino groups that can be used for further crosslinking or polyurethane production. The synthesis of lignin-based epoxy resins, their properties and potential applications are sufficiently covered in the literature elsewhere.^68^

### Ethylenediamine pretreatment

In several studies, it has been reported that ethylenediamine (EDA) pretreatment of lignocellulosic biomass is an efficient procedure for deconstructing the lignin–carbohydrate complex structure to enhance the carbohydrate and bioethanol production.^69–74^ EDA pretreatment allows to enhance the solubility of lignin and improve the lignin removal from the biomass. In general, for this procedure the lignocellulosic biomass is mixed with EDA and heated, followed by cooling of the reaction mixture. After the procedure, the obtained solids can be separated by filtration and subjected to ethanol precipitation to isolate the aminated lignin. The benefit of this approach is that unreacted EDA can be recovered from the filtrate by evaporation and recycled. The amount of added EDA, temperature, residence time and, therefore, the nitrogen content typically varies based on the exact procedure applied.

**Scheme 14:**
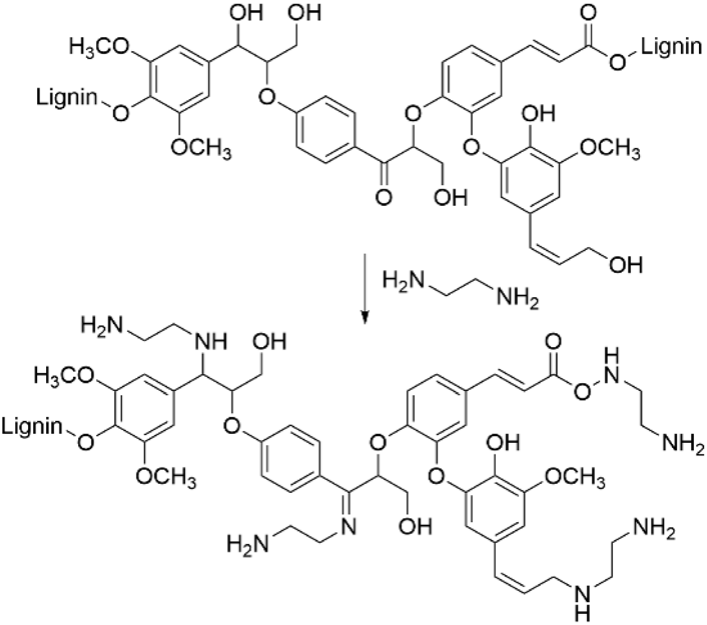
Lignin amination by ethylenediamine pretreatment methodology.

It has been shown that after EDA pretreatment, a major part of the acid-insoluble lignin in lignocellulosic biomass is converted to acid-soluble lignin. As a result of the EDA pretreatment, amination of α-hydroxyl groups in β-O-4 units was detected with 16.70% nitrogen content.^73^ In other work, EDA pretreatment of alkaline lignin allowed to obtain aminated lignin with the nitrogen content reaching up to 19.6 wt%. Based on the appearance of new groups detected by NMR spectroscopy, the amination pathway involving different lignin units was proposed (Scheme 14).^74^

In addition, lignin depolymerization and formation of low-molecular weight products was observed. Thus, EDA pretreatment method allows to treat lignocellulosic feedstocks, resulting in the formation of aminated lignin with enhanced solubility and reduced condensation, that can be beneficial for further utilization of lignin in high value-added applications.

### Silylation and amination

**Scheme 15:**
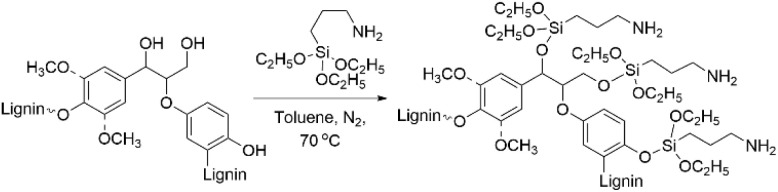
Lignin amination *via* silylation with aminosilanes.

Amino groups can also be introduced to lignin structures by silylation of the lignin hydroxyl groups with aminosilanes (Scheme 15).^75–77^ Silylation of lignin has been investigated in several studies, resulting in successful introduction of silyl groups to lignin structures.^60,78,79^ Daugaard *et al.* have demonstrated that amino groups can be introduced to lignin by ring-opening silylation with cyclic aza-silanes for producing lignin-based curing agents for epoxy resins.^75^ Kim *et al.* performed one-step silylations of lignin with (3-aminopropyl) triethoxysilane.^76^ After the reaction, the nitrogen content in the modified lignin reached up to 6.10 wt%. The introduction of aminosilane functionalities to lignin structure led to increase in the molecular weight, enhanced thermal stability and solvent-resistant properties of the obtained modified lignin. In other work, Kim *et al.* carried out the silylation of lignin with three aminosilanes containing primary, secondary and tertiary amines, producing aminated lignin containing different types of amino groups.^77^ For primary aminosilane, the tendency to undergo self-condensation was observed, resulting in the formation of silicon network in the lignin studied. When tertiary aminosilane was used, crosslinking processes in the lignin were recorded. The nitrogen content in lignin containing primary amino groups was the highest, reaching 8.29 wt%.

### Amidation-hydrolysis

**Scheme 16:**
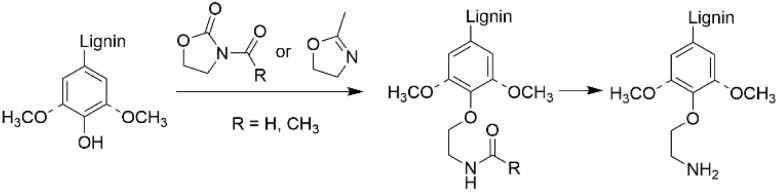
Lignin amination by two-step amidation-hydrolysis reaction.

Heterocyclic, nitrogen containing 2-oxazolidinones and oxazolines can also be used for introducing amino groups to lignin structures by two-step amidation-hydrolysis approach (Scheme 16).^80,81^ Renneckar *et al.* developed a lignin amination procedure using 2-oxazolidinone both as a solvent and as a reagent. This method allows to obtain lignin containing primary and secondary amino groups with nitrogen contents reaching up to 7.97 wt%.^80^

In recent work, Avérous *et al.* described a two-step amination procedure to obtain aminated lignin using two heterocyclic compounds, 2-methyl-2-oxazoline and *N*-acetyl-2-oxazolidinone.^81^ The procedure introduces primary hydroxyl groups to lignin by utilizing the phenolic hydroxyl groups, providing aminated lignin with low molecular weight and high solubility. The nitrogen content in the aminated lignin reached up to 3.5 mmol g^−1^ (4.9 wt%).

## Discussion and conclusion

In recent years, development of lignin valorization methodologies has emerged as a promising alternative for producing new bio-based materials and chemicals. The number of studies on lignin modification and analysis have expanded significantly, providing better understanding of lignin reactivity and the possible valorization pathways. As an important example, amination of lignin hydroxyl groups provides a highly promising approach towards lignin functionalization and the production of value-added nature-based products, including advanced polymeric materials and fine chemicals, that can be used as building blocks also for the synthesis of biologically active compounds.

It has been shown in several studies on lignin model compounds that catalytic amination methodologies allow to obtain a wide range of aromatics, bearing different N-containing functionalities. The reactions commonly undergo several subsequent steps, including dehydrogenation, bond cleavage and reductive amination, resulting in the formation of benzylamines, amides, imines, heterocyclic and other nitrogen containing compounds with a wide range of potential applications. Several catalytic procedures successfully performed on the model compounds have also been applied to extracted lignin for degradation towards monomeric aminated products, demonstrating the significant potential of this valorization pathway. However, only in few studies the developed procedures have been applied to extracted lignin demonstrating applicability towards direct valorization. The most common model compounds used for developing procedures for lignin valorization are β-O-4 ethers imitating the most abundant linkage in native or extracted lignin. Use of the model compounds provides better understanding of lignin reactivity or, more specifically, the reactivity of lignin units that the model compounds represent. However, at the same time, the model compounds lack the structural complexity of lignin that can significantly affect the reactivity. In catalytic reactions the interaction of a catalyst with other lignin functionalities not considered during studies on the model compounds can lead to unknown side reactions and significantly affect the outcome of the catalytic modification. In addition, the impurities in lignin feedstock can cause catalyst poisoning and deactivation.^82^ Using more complex oligomeric model compounds bearing multiple lignin linkages or mixtures of monomeric and dimeric model compounds could create and simulate reaction conditions that would provide more complete understanding of the reactivity of actual lignin, and applicability of the tested conditions towards real lignin valorization.

Another aspect that should be considered is that in technical lignins obtained after wood biomass processing, the content of β-O-4 units decreases significantly. In lignin directly extracted from Eucalyptus wood the content of β-O-4 units has been reported as 55%, while being 38% in sulfonate lignin, 8% in alkaline lignin, and only 2% in Kraft lignin.^83^ The decrease of β-O-4 units content could become a limitation for application of catalytic procedures to technical lignins.

For the amination of technical lignins, the approaches targeting phenolic and aliphatic hydroxyl groups can be used. Mannich reaction is a straightforward procedure, successfully applied for amination of different types of industrial lignins. However, the amino groups introduced to lignins by this method are predominantly tertiary amines, restricting the potential application of the obtained product. Lignin amination *via* preliminary epoxy functionalization, in turn, allows to introduce primary and secondary amino groups to lignin. With this method, it is important to prevent crosslinking reactions between the epoxy groups and the primary/secondary amines leading to formation of tertiary amines. Silylation with aminosilanes, in turn, introduces amino groups with different composition, depending on the exact aminosilanes used for the modification. With this method, lignin bearing multi-layer crosslinked structures containing more amino groups can be formed due to the ability of silanes to undergo self-condensation and to form polysiloxanes. Amidation-hydrolysis allows to obtain aminated lignin containing primary hydroxyl group *via* a two-step reaction. This method utilizes non-toxic chemicals and results in the formation of highly soluble low molecular weight lignin. EDA pretreatment is another simple method for generation of aminated lignin that can be applied directly to lignocellulosic biomass, extracted lignin and alkaline lignin. This method allows to obtain aminated lignin with high nitrogen content, containing both primary and secondary amino groups. A summary of the lignin amination procedures described in this review are presented in Table 1.

**Table 1:** Summary of lignin amination methods

Amination procedure	Reagent	Type of lignin	Amino group composition	Highest reported nitrogen content, wt%
Mannich reaction	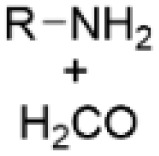	Alkali, Kraft, soda and organosolv lignin	Tertiary	10.13
Amination *via* preliminary epoxy functionalization	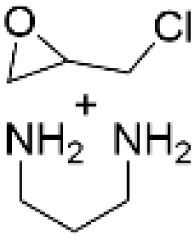	Alkali lignin	Primary and secondary	6.95
Ethylenediamine pretreatment	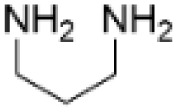	Lignocellulosic biomass, extracted lignin and alkaline lignin	Primary and secondary	16.70
Silylation with amino silanes	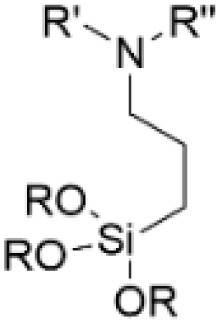	Kraft lignin	Primary, secondary and tertiary	8.29
Amidation-hydrolysis	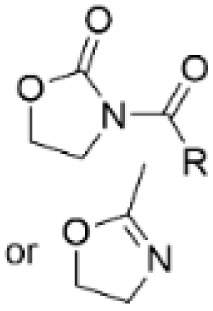	Technical lignin (Kraft, soda and organosolv)	Primary	7.97

Thus, a wide range of different methodologies can be used for the synthesis of lignin-based aminated products.

Lignin amination can be potentially implemented into the early-stage catalytic conversion of lignin “lignin-first” strategy.^84^ The amination of model β-O-4 compounds in the presence of several catalytic systems commonly undergoes dehydrogenation and bond cleavage, resulting in the formation of acetophenones that further react with the aminating reagent. Instead of the model β-O-4 compounds, these catalytic systems can be used either for amination of vanillin or other lignin fractionation products or for direct fractionation – amination of organosolv lignin containing β-O-4 units to produce nitrogen containing fine chemicals.

Another approach for lignin valorization involves the amination of ethanosolve lignin under mild conditions, with preservation of its polymeric structure, to produce bio-based polymeric materials.

Technical lignins (Kraft, alkaline, soda) have a higher content of free phenolic hydroxyl groups (3.3–4.3 mmol g^−1^) that can be targeted for chemical modification and incorporating amino groups into lignin structures.

From the biorefinery point of view, organosolv process allow to obtain pure lignin with high content of aliphatic hydroxyl groups with low molecular weight, that can be further utilized for chemical modification or fractionation. However, the extraction process involves the use of toxic and flammable organic solvents that can only be recovered at high energy cost. “Lignin-first” strategy, including the reductive catalytic fractionation approach, can be used for production of lignin-based fine chemicals. The reductive conditions of the process allow to prevent lignin condensation and to reach high selectivity and yields. The main disadvantages of this approach are the high cost of the catalysts and their deactivation, and the formation of complex mixtures of products that require separation. Development of robust and efficient catalytic systems, reducing the solvent and energy demands, and integrating these processes into biorefinery environments would facilitate efficient and sustainable lignin utilization.

Depending on the source of lignin and its composition, different methods can be used for valorization towards valuable nitrogen containing products. Lignin-derived monomers or organosolv lignin can be successfully converted into primary, secondary and tertiary amines, including heterocyclic products with potential biological activity. Technical lignins can be converted into various materials with a broad range of potential applications, including fertilizers, bio-based polyurethanes, curing agents for epoxy resins, and nanoparticle formation. Thus, despite the complexity and heterogeneity of lignin as starting material, promising methods for lignin valorization have been developed over the recent years, proving excellent prospectives for successful future valorization towards value-added products.

## Author contributions

Veronika Badazhkova: writing – review & editing, writing – original draft. Risto Savela: writing – review & editing, supervision. Reko Leino: writing – review & editing, supervision, conceptualization.

## Conflicts of interest

The authors declare that they have no known competing financial interests or personal relationships that could have appeared to influence the work reported in this paper.

## Data Availability

No primary research results, software or code have been included and no new data were generated or analysed as part of this review.
